# Supplementary material for: Twist-angle engineering of excitonic quantum interference and optical nonlinearities in stacked 2D semiconductors
Source: Nat Commun. 2021 Mar 10;12:1553. doi: 10.1038/s41467-021-21547-z (PMC7946969; doi:10.1038/s41467-021-21547-z)
Supplement: Supplementary file 1 — Supplementary Information [file 41467_2021_21547_MOESM1_ESM.pdf]

Supplementary Information for

Twist-angle engineering of excitonic quantum interference and optical nonlinearities in stacked 2D semiconductors

Lin *et al.*

Supplementary Note 1

We calculate the band structure of monolayer WSe_2 in Fig. 2a, using the full-potential linearized augmented plane-wave method as implemented in the Wien2k package[1]. We use the Perdew-Burke-Ernzerhof (PBE) exchange-correlation functional[2]. Self-consistency is achieved using a Monkhorst-Pack $\boldsymbol{k}$-grid of 15×15×1 with convergence criteria of 10^{-6} e for the charge and 10^{-6} Ry for the energy. The wave functions are expanded in atomic spheres with orbital quantum numbers up to 10 and the plane-wave cutoff multiplied by the smallest atomic radii is set to 8. Spin-orbit coupling is included fully relativistically for core electrons, while valence electrons are treated within a second variational procedure with the scalar-relativistic wave functions calculated in an energy window up to 5 Ry. The experimental lattice parameters of in-plane lattice constant a = 3.286 Å and a distance $d_{Se\text{-}Se}$ = 3.34 Å between the two Se planes are adopted from Ref. [3] and a vacuum spacing is set to 20 Å to avoid interactions between slabs.

To calculate the partial charge density for states as shown in Fig. 2b, first-principles calculations are performed using the Vienna *ab-initio* simulation package (VASP)[4,5], based on state-of-the-art density functional theory (DFT). The projector augmented-wave potential is used with W $5p^6 5d^4 6s^2$ and Se $4s^2 4p^4$ valence states. Spin-orbit coupling is not included. The generalized gradient approximation (GGA) in the PBE revised for solids (PBEsol) is used for the exchange-correlation functional[2]. Based on the convergence tests, we use a kinetic energy cutoff of 500 eV and a Γ-centered 12×12×1 $\boldsymbol{k}$-mesh to sample the electronic Brillouin zone. The convergence parameters for structural relaxations include an energy difference within 10^{-6} eV and a Hellman-Feynman force within 10^{-4} eV/Å. We maintain the interlayer vacuum spacing larger than 15 Å to eliminate interactions between adjacent layers.

The interlayer distances of WSe_2 homobilayers, presented in the Supplementary Fig. 5, are calculated using the Wien2k package with the PBE exchange-correlation functional. The van der Waals interaction is included via the D3 correction[6]. We find the optimized interlayer distance without accounting for spin-orbit coupling. For the 0° (3R) and 60° (2H) stacking configurations of bilayer WSe_2, we used the same convergence parameters as in monolayer WSe_2. For the 21.79° and 38.21° twisted bilayer WSe_2, we consider a Monkhorst-Pack $\boldsymbol{k}$-grid of 12×12×1, and convergence criteria of 10^{-5} e for the charge and 10^{-5} Ry for the energy. The plane-wave cutoff multiplied by the smallest atomic radii is set to 7 and the vacuum spacing is set to 20 Å.

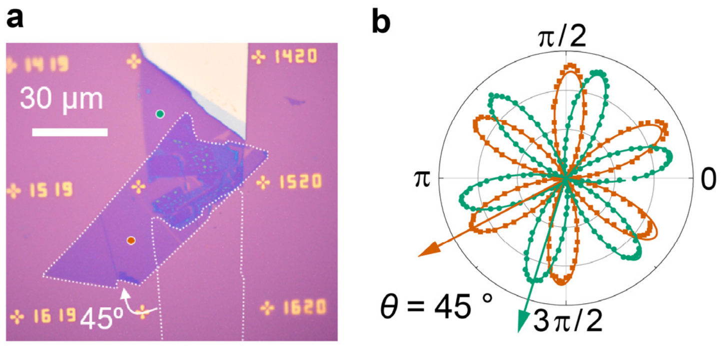

Supplementary Figure 1 | Fabrication and determination of twist angle in artificially stacked bilayer WSe_2. a, Microscopic image of a representative twisted bilayer WSe_2 with an angle of 45° fabricated by subsequently stamping two segments of one monolayer of WSe_2. **b**, The SHG intensity copolarized with the incident laser as a function of crystal angle, measured on two segments of the flake after stamping (orange, green). The measurement positions on the flake are illustrated as orange and green dots in (a).

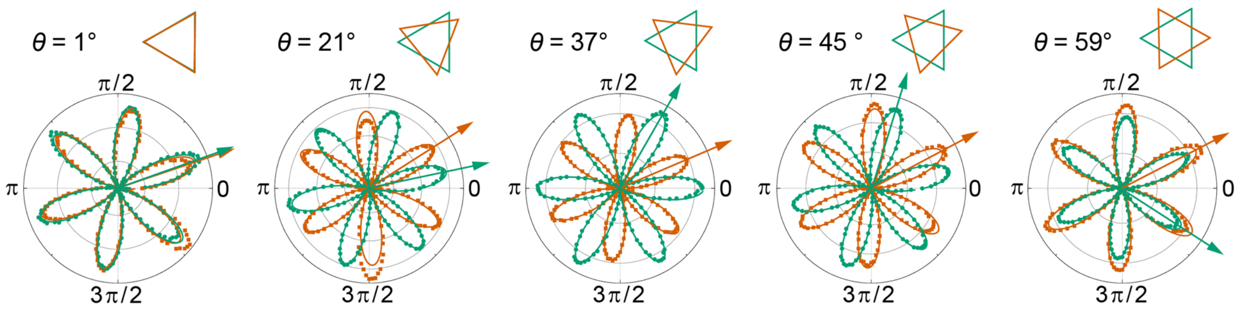

Supplementary Figure 2 | Determination of the twist angle in artificially stacked bilayer WSe_2 in Fig. 2e using the SHG polarization as described in the Supplementary Fig. 1.

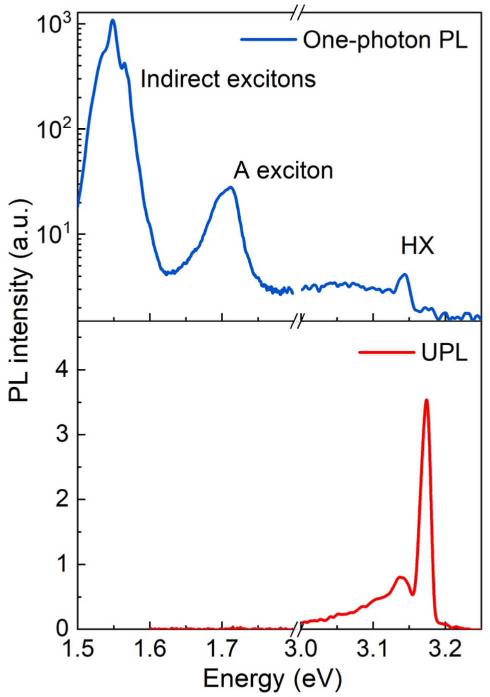

Supplementary Figure 3 | One-photon PL and UPL of the HX from hBN-encapsulated bilayer WSe_2 on a sapphire substrate. The one-photon PL of the HX was measured by exciting the bilayer WSe_2 with a 325 nm (3.81 eV) continuous-wave Helium-Cadmium laser. An aluminum reflective objective (36×, NA = 0.5, Beck Optronic Solutions) was used to focus the laser and collect the PL signals, and a D-shape UV-enhanced aluminum mirror was used in place of the beam splitter.

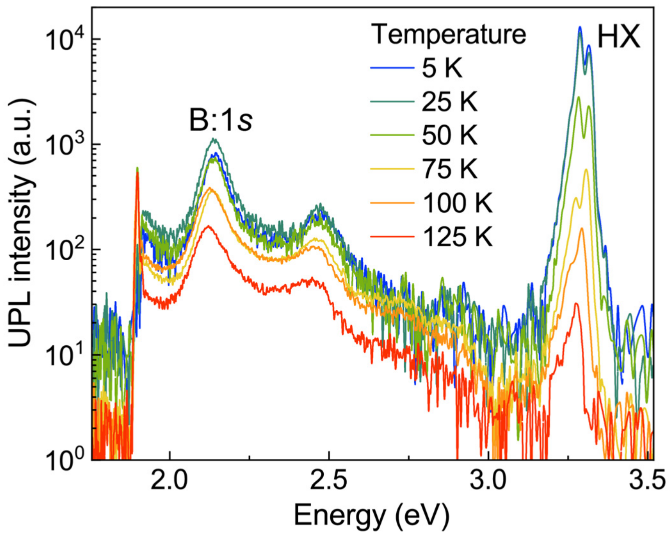

Supplementary Figure 4 | Temperature dependence of the UPL of 40° twisted-bilayer WSe_2. The HX UPL intensity decreases with increasing temperature and persists above 125 K. The UPL of the HX is ten times stronger than that of the B:1*s* exciton at 5 K, but five times weaker at 125 K. This decrease of the HX UPL intensity likely results from both thermal broadening of the A-exciton transition, which limits resonant pumping by UPL, and an increase in non-radiative decay of the HX with increasing temperature. The UPL was measured by pumping the sample with a CW laser tuned to typically 720 nm with a power of 50 µW. To follow the shift of the A-exciton resonance with temperature, the laser wavelength was tuned to 724 nm for the 100 K measurement and to 727 nm at 125 K.

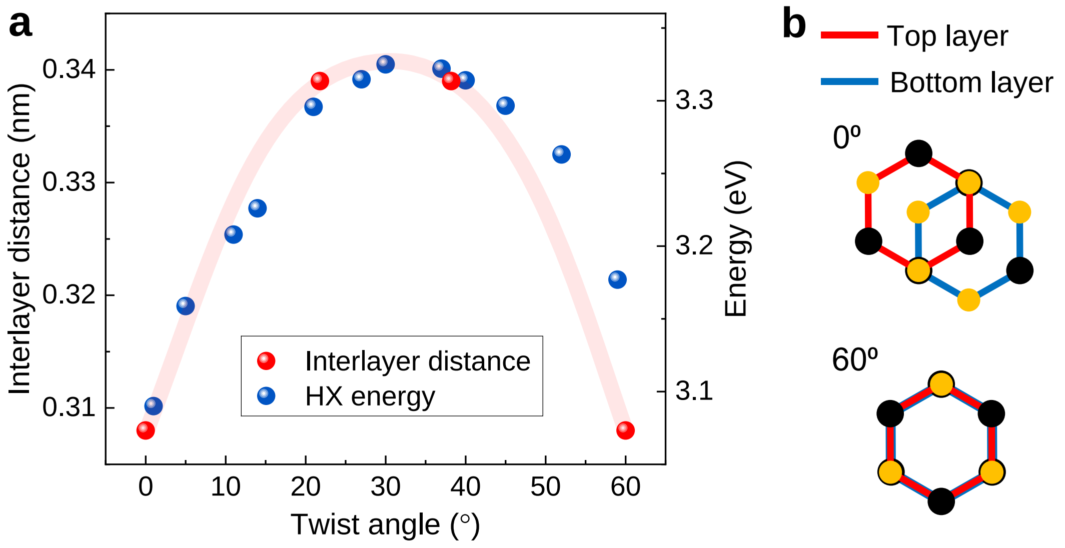

Supplementary Figure 5 | Correlation between interlayer distance and high-lying exciton energy. a, The dependence of the calculated interlayer distance and the experimental HX energy on twist angle of WSe_2 homobilayers. The red line serves as a guide to the eye. **b,** Illustration of the 0º (3R) and 60º (2H) configurations used in the calculation of interlayer distances.

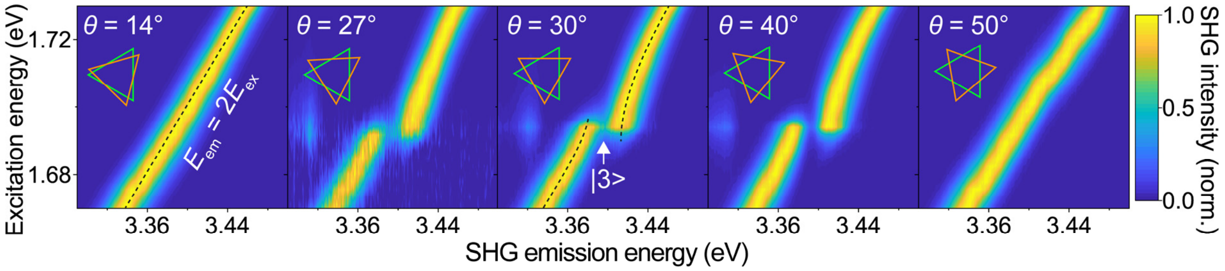

Supplementary Figure 6 | Experimental twist-angle dependence of quantum interference in SHG from bilayer WSe_2 at 5 K. Normalized SHG intensity as a function of emitted photon energy (horizontal axis) and central photon energy of the pulsed excitation laser (vertical axis). The corresponding stacking angles are indicated in the insets.

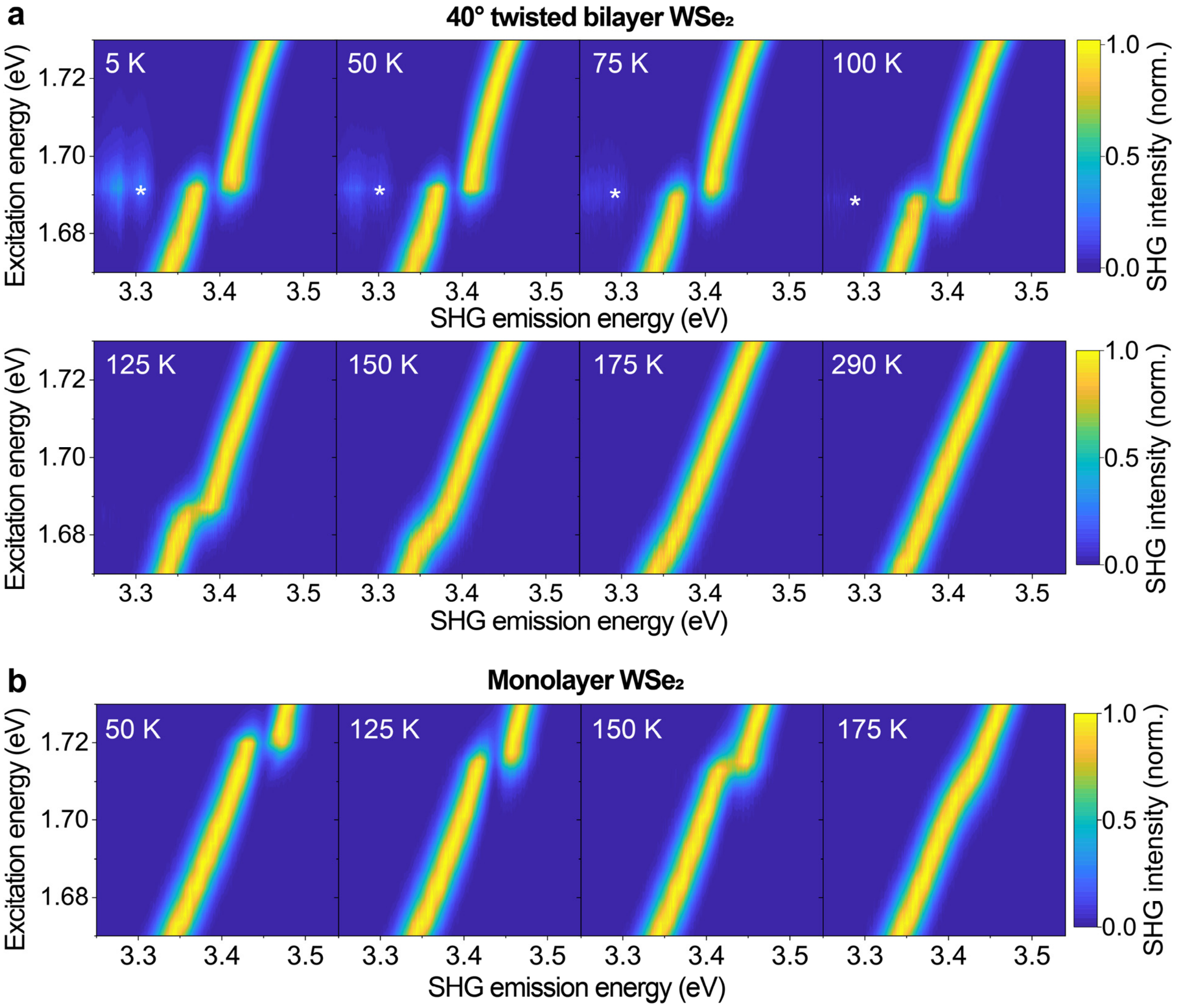

Supplementary Figure 7 | Temperature dependence of quantum interference in SHG of 40° twisted-bilayer WSe_2 (a) and monolayer WSe_2 (b). The anticrossing feature of the quantum interference persists up to a temperature of 125 K for twisted-bilayer WSe_2 and up to 150 K for monolayer WSe_2. For twisted-bilayer WSe_2, the intensity of the HX UPL (marked by asterisks) decreases with increasing temperature. The measurements were carried out by scanning the excitation wavelength of an 80-fs pulsed laser from 715 nm to 745 nm with 1 nm steps while keeping the laser power at 1 mW. A 1200 grooves/mm grating was used and the integration time of the spectrometer was set to 10 s.

	Twist-angle susceptibility	Sources
HX (WSe_2)	~8.1 meV/°	This work
A-exciton (K-K exciton, WSe_2)	~0.8 meV/°	This work
K-Λ exciton (WSe_2)	< 3 meV/°	Ref. 8
Interlayer exciton ($MoSe_2/WS_2$)	~1.8 meV/°	Ref. 9
Interlayer exciton ($MoSe_2/WSe_2$)	~1.6 meV/°	Ref. 10

Supplementary Table 1 | Comparison of the susceptibility of the exciton transitions to twist angle.

Supplementary References

1 Blaha, P. *et al.* WIEN2k: An APW+lo program for calculating the properties of solids. *J. Chem. Phys.* **152**, 074101 (2020).

2 Perdew, J. P. *et al*. Restoring the density-gradient expansion for exchange in solids and surfaces. *Phys. Rev. Lett.* **100**, 136406 (2008).

3 Kormányos, A. *et al.* k·p theory for two-dimensional transition metal dichalcogenide semiconductors. *2D Mater.* **2**, 022001 (2015).

4 Kresse, G. & Furthmüller, J. Efficient iterative schemes for ab initio total-energy calculations using a plane-wave basis set. *Phys. Rev. B* **54**, 11169-11186 (1996).

5 Kresse, G. & Furthmüller, J. Efficiency of ab-initio total energy calculations for metals and semiconductors using a plane-wave basis set. *Computational Materials Science* **6**, 15-50 (1996).

6 Grimme, S., Antony, J., Ehrlich, S. & Krieg, H. A consistent and accurate ab initio parametrization of density functional dispersion correction (DFT-D) for the 94 elements H-Pu. *J. Chem. Phys.* **132**, 154104 (2010).

7 Lin, K.-Q. *et al.* Bright excitons with negative-mass electrons. *arXiv:*2006.14705 [cond-mat.mes-hall].

8 Merkl, P. *et al.* Twist-tailoring Coulomb correlations in van der Waals homobilayers. *Nat. Commun.* **11**, 2167 (2020).

9 Alexeev, E. M. *et al.* Resonantly hybridized excitons in moiré superlattices in van der Waals heterostructures. *Nature* **567**, 81 (2019).

10 Kunstmann, J. *et al.* Momentum-space indirect interlayer excitons in transition-metal dichalcogenide van der Waals heterostructures. *Nat. Phys.* **14**, 801 (2018).
